# A national school-based health lifestyles interventions among Chinese children and adolescents against obesity: rationale, design and methodology of a randomized controlled trial in China

**DOI:** 10.1186/s12889-015-1516-9

**Published:** 2015-03-03

**Authors:** Yajun Chen, Lu Ma, Yinghua Ma, Haijun Wang, Jiayou Luo, Xin Zhang, Chunyan Luo, Hong Wang, Haiping Zhao, Dehong Pan, Yanna Zhu, Li Cai, Zhiyong Zou, Wenhan Yang, Jun Ma, Jin Jing

**Affiliations:** Department of Maternal and Child Health, School of Public Health, Sun Yat-sen University, Guangzhou, China; Institute of Child and Adolescent Health, School of Public Health, Peking University, Beijing, China; Department of Maternal and Child Health, School of Public Health, Central South University, Changsha, China; School of Public Health, Tianjin Medical University, Tianjin, China; Shanghai Municipal Center for Disease Control and Prevention & Shanghai Institutes of Preventive Medicine, Shanghai, China; Chongqing Medical University, Chongqing, China; Ningxia Medical University, Ningxia, China; Liaoning Health Supervision Bureau, Shenyang, China

**Keywords:** Chinese children and adolescent, School-based obesity intervention, Multi-centered randomized controlled trial

## Abstract

**Background:**

The prevalence of obesity among children and adolescents has been rapidly rising in Mainland China in recent decades, both in urban and rural areas. There is an urgent need to develop effective interventions to prevent childhood obesity. Limited rigid data regarding children and adolescent overweight prevention in China are available. A national random controlled school-based obesity intervention program was developed in the mainland of China.

**Methods/Design:**

The study was designed as a national multi-centered cluster randomized controlled trial involving more than 70,000 children and adolescents aged 7–18 years from 7 provinces in China. In each center, about 12–16 primary and secondary schools, with totally at least 10000 participants were randomly selected (Primary: Secondary = 1:1). All of the selected schools were randomly allocated to either intervention or control group (Intervention: Control = 1:1).The multi-components school-based and family-involved scheme was conducted within the intervention group for 9 month, while students in the control group followed their usual health practice. The intervention consisted of four components: a) Create supportive school and family environment, b) Health lifestyles education and related compulsory physical activities, c) Instruct and promote school physical education, d) Self-monitor obesity related behaviors. Four types of outcomes including anthropometric, behavioral, blood chemical and physical fitness were measured to assess the effectiveness of the intervention program.

**Discussion:**

This is the first and largest multi-centered school-based obesity intervention program with the consideration of geographical and social-demographic characteristics of the rapidly increased obesity prevalence of Chinese children and adolescent. The intervention is based on Social Cognitive Theory and Social-Ecological Model of Health, and follows a stepwise approach guided by PRECEDE-PROCEED (P-P) Model and Intervention Map. The results of and lesson learned from this study will help guide future school-based national childhood obesity prevention programs in Mainland China.

**Trial registration:**

January 22, 2015; Registration number: NCT02343588

## Background

Childhood obesity has become a global epidemic [1]. During the past couple of decades, China has experienced rapid socio-economic and nutritional transitions [2,3]. Along with these lifestyle changes, the prevalence of overweight among children and adolescent rose from 6.3% in 1991 to 8.8% in 2000, and to 17.1% in 2011; besides, younger cohorts are now experiencing comparatively higher body mass index (BMI) at earlier ages than ever before [4]. Overweight and obese youth are more likely to suffer from a variety of physical and psychosocial problems [5,6]. A latest review suggested that obesity youth are more likely than non-obesity peer to be a target of metabolic disease, internalizing disorders and attention-deficit hyperactivity disorder [7]. Moreover, overweight in early childhood has been shown to track to adulthood in one-third to one half of cases [8], where it becomes associated with an increased prevalence of chronic disease [9]. Therefore it is urgent to do effective interventions to reduce childhood overweight and obesity.

Since the 1990s, a number of intervention studies for the prevention or treatment of overweight among children and adolescents has been conducted in China. The results of a systematic review have revealed that most of these studies addressing obesity prevention are of low methodological rigor. Although many of the studies stated that the intervention and control of allocation were randomized, no description of the method of randomization was reported. Neither was there any evidence that assessors were blinded to the study intervention. Most of the studies didn’t report that they sought informed consent prior to commencement of the trials [10]. Interventions focusing on only overweight/obesity children limit their conclusions to be deduced to common population [11]. Moreover, the evaluation of the efficacy of the study interventions was limited by the lack of information on loss to follow-up [12,13]. In terms of the type of intervention, besides physical activity and diet health education, little research included the modification of school and family environment, which are key factors influence the intervention effect [14]. In spite of the limited good quality data on which to draw conclusions about obesity prevention in children and adolescents in China, recent findings from primary school-based intervention programs have delivered some encouraging findings [15–17]. Schools are potentially important channels of intervention because they offer access to large populations of children, often with mixed socioeconomic background, and provide the opportunity to institutionalize programs in communities [10]. However, limited number of school-based intervention programs for teenagers and whether the intervention programs would be successful when expand in large scale (from more regions to national-wide) remains unclear in China [18]. Therefore, we were conducting a school-based intervention study in 7 provinces with more than 70,000 students in China from the September of 2013 to the June of 2014.

The Health Lifestyles Interventions is a multi-center random controlled school-based intervention for children and adolescent in China, which includes nutrition, physical activity (PA) and school environment intervention as well as providing several opportunities for parental engagement. We hypothesized that the comprehensive interventions will lead to improvements in diet and PA information, motivation and behaviors, further preventing children and adolescent obesity and related cardiovascular risk factors.

### Aims

The aim of this multi-centered cluster randomized controlled trial (RCT) is to determine the effectiveness and cost-effectiveness of the Health Lifestyles Interventions in preventing overweight and obesity in Chinese school children and adolescent.

### Specific objectives

1. To assess the effectiveness of the Health Lifestyles Interventions, in children and adolescent aged 7–18 years, by comparing in intervention and control schools:
  **Primary outcomes:** Change in the prevalence of overweight and obesity
  **Secondary outcomes:**
    - Anthropometric outcomes: waist circumference, hip circumference, blood pressure and skin fold thickness;
    - Behavioral outcomes: dietary, sedentary or PA behaviors and their determents (e.g. children’s or parental knowledge, beliefs and attitudes, parental BMI, PA and eating behaviors, school environment);
    - Blood chemical outcomes: fasting plasma glucose, fasting triglycerides, total cholesterol, high-density lipoprotein cholesterol (HDL-C), low-density lipoprotein cholesterol (LDL-C);
    - Physical fitness outcomes: standing-board jump, 50 meters speed run, 50 meters × 8 shuttle run (primary students) and run 800/1000 meters (secondary school students).

2. To conduct a process evaluating by qualitative interviews and other methods to explore the way the interventions worked (that is, how it was delivered, taken up and experienced, and what the behavioral mediators of change are).

## Methods/Design

### Design

The study is designed as a national multi-centered cluster randomized controlled trial involving more than 70,000 participants from Liaoning, Tianjin, Ningxia, Shanghai, Chongqing, Hunan, and Guangdong to determine the effectiveness of the Health Lifestyles Interventions in preventing Chinese children and adolescent obesity at 9 month. Standardized and uniform research protocol will be applied in all intervention schools in the 7 provinces.

**Figure 1:**
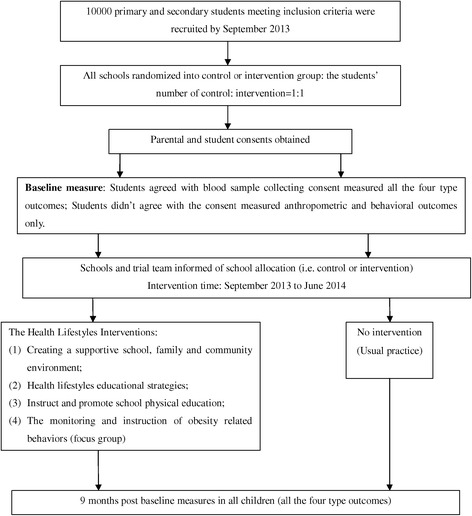
**Shows the flow of participants through the trial in each center.** In each center, about 10000 eligible primary and secondary (primary: secondary = 1:1) students were recruited, then were randomly allocated to either intervention or control group. Parental and student consents obtained. Baseline measurements were taken by assessors at September 2013. The multi-components school-based and family-involved scheme was conducted within the intervention group for 9 month. Post baseline measurements were taken by assessors at June 2014, four types of outcomes including anthropometric, behavioral, blood chemical and physical fitness were measured at baseline and post baseline to assess the effectiveness of the intervention program.

Figure 1 shows the flow of participants through the trial in each center.

### Participants

**Figure 2:**
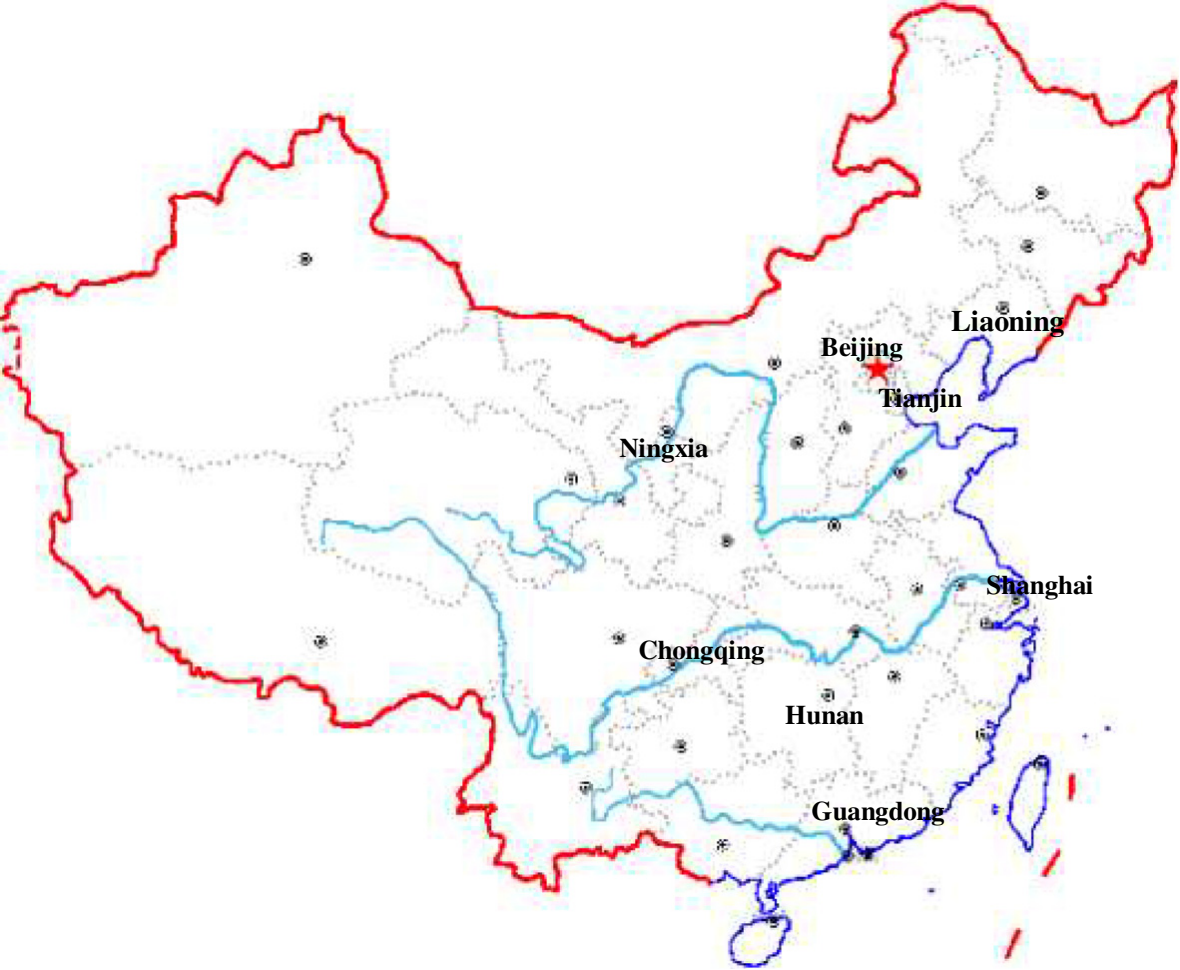
**The geographic distribution of involved provinces and municipalities.** This study was designed to be multi-center school-based cluster randomized controlled obesity intervention trial. Liaoning, Tianjin, Ningxia, Shanghai, Chongqing, Hunan, and Guangdong were involved in it.

Liaoning, Tianjin, Ningxia, Shanghai, Chongqing, Hunan, and Guangdong were involved in this multi-center school-based obesity intervention project (Figure 2). About 12–16 primary and secondary schools, with totally at least 10000 students aged 7–18 years, were enrolled in each center (The students number: primary school: secondary school = 1:1; intervention: controlled = 1:1; urban: rural = 1:1). Students in the last year of primary and secondary school (grade 6, 9 and 12) were not contacted due to their study load.

### Sample size calculations

The target sample size was calculated to detect a difference of 10% obesity prevalence between the intervention and control groups (defined by using WGOC criteria) [19] at 9 months, the minimum number required would be 420 of each grade in each center, the sample size of 70000 students from 70 schools located in7 centers has 90% power to detect a significant difference in obesity prevalence. Statistical significance is set at 5% (two-sided).

### Ethical approval and consent

This study was approved by the Ethical Committee of the Peking University. All participant students and their parents signed informed consents voluntarily.

### Randomization and blinding

Multistage cluster sampling was used. The random sequence of schools will be computer generated and stratified by (1) school district; (2) school grades (primary or high) and (3) school size in each center. All of the students in the selected schools of different grades, namely, grades 1 to 12, were invited to participate in the survey. After a school is confirmed to be eligible and both parents and children written informed consent have been obtained, it will be randomly assigned to intervention or control group. Intervention or control schools in the same stratification were allocated using simple random sampling method. Randomization will be performed by a member of staff who is not involved with the trial immediately after all schools have been recruited according to the standard.

### Recruitment process

The principles (school head) of selected schools were sent an invitation letter, an information sheet, and a presentation containing study details. Then all of the selected school principles were attended a face-to-face appointment to discuss the study. During the study meeting with the principal, the study aims, proposed methods, study procedures were discussed. With the principal’s permission, the parent and student consent forms including physical examination and blood sample collection were given to each student to bring home. The children were asked to return the consent forms to the school if they and their parents were willing to participate. Overall, the two consent forms were consisted of three sections. The first section gave permission for the child to participate in the data collections, including the enrollment of anthropometric and physical fitness measurements and the response of questionnaires. The second section gave permission for a blood sample. The third section gave permission for the child to participate in the obesity related intervention strategies.

### Intervention

**Figure 3:**
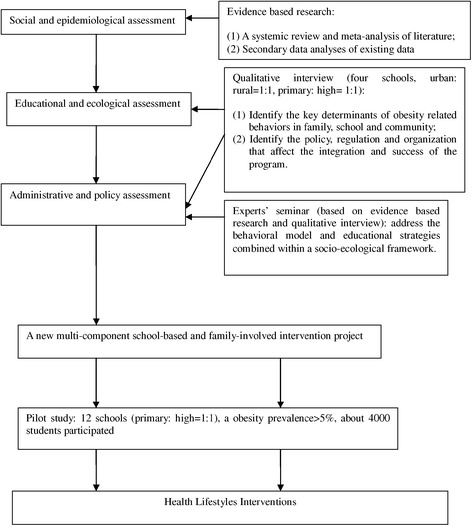
**The Process of the intervention strategies design.** Health Lifestyles Interventions is a multi-components school-based and family-involved scheme. It was developed through the process: **a)** Social and epidemiological assessment, **b)** Educational and ecological assessment, **c)** Administrative and policy assessment, **d)** Pilot study.

Health Lifestyles Interventions is a multi-components school-based and family-involved scheme which takes place over nine month and aims to deliver a general healthy lifestyle message encouraging a healthy energy balance. Literature review, qualitative interview and piloting has demonstrated that it is useful to focus on changing specific behavior patterns related to energy intake and expenditure, such as a decrease in the consumption of sweetened fizzy drinks; increasing the consumption of vegetables; increasing the ratio of healthy to unhealthy snacks; a reduction in sedentary behavior activities; and at least one hour moderate to vigorous PA (Figure 3). Firstly, *creating a supportive school and family environment,* including learn about what is known about obstacles and success factors that influence the development and implementation of these programs for the target group, setting the foundation for successful delivery of subsequent components; Secondly, the intensive *Health lifestyles educational strategies* involving health education lessons and related compulsory activities towards children and parents; Thirdly, *instruct and promote school physical education*; Fourthly, set up *the monitoring and instruction of obesity related behaviors* based on the messages learned before (Table 1).

**Table 1:** **Interventions, change targets, concrete techniques and the periods and agent of delivery**

**Interventions**	**Change targets**	**Concrete techniques**	**Intervention periods**	**Agent of delivery**
Creating supportive school and family environment	Establish relationships with schools, children and families	Provide necessity exercise facilities and ensure at least one hour exercise time every school day	Whole intervention periods	School manager
		Provide information on obesity preventive (through poster, broadcast and website) to students and parents		Project members
	Create a good environment for physical activity and health dietary	Create a health school dietary environment: provide boiled water; no sweetened fizzy drinks in class and school shops.		Class teachers and school shop sellers
		Regulate school exercise and dietary institution according to national standards		School manager
Health lifestyles educational strategies	Strengthen relationships with schools, children and families	Student health education (lecture, theme class meeting)	30 min/week Six times Two times (60 minutes)	Project members
	Increase knowledge	Student health education activities		Health education teacher
	Increase social support(school, peer and family)	Parental health education lecture		School doctor
Instruct and promote school physical education	Standardization and rationalization physical education	Design and revise physical education lesson plan according to model plan	Whole intervention periods	Project members
		Design and revise physical education activities according to physical activity prescription		School physical education teacher
The monitoring and instruction of obesity related behaviors	Increase awareness of own behavior	Physical activity and dietary behavioral log	Once a week	Student
	Strengthen health lifestyle knowledge	Measure weight and height regularly	Once a month	School doctor
	Increase self-efficacy for change	Parental monitor (feedback regularly to parents)		

### Intervention quality control

A manual (Students Obesity Prevention and Health Promotion Instruction Manual) have been developed to accompany this complex intervention. The intervention manual describes the definition, prevalence, influence, risk factors and screening standard of students’ obesity in China. Then it includes the definitions of the concrete techniques (frequency and duration) and a checklist that has been created to ascertain how and to what extent the intervention has been delivered as specified. It also details the training and duty of each delivery personnel (Project members, school manager, class teachers, school doctor and students). Some successful models are added to learn and refer. Moreover, a nutrition status self-assessment turn plate was created for all of the participants.

Before the project all the intervention school doctors, health education teachers and physical education teachers were trained by the professional members of the project. Specialized members were arranged to supervise the intervention schools throughout the program. All of the advocate and educational materials were developed and provided by the project team.

All of the intervention schools would be supervised twice during the intervention periods by project managers. In addition to supervision, existing school practices will be carefully characterized and recorded using a predetermined checklist of possible school level mediators of childhood obesity. School activities which may affect diet and/or PA behaviors (for example, number hours of PA, health school status, personal, social, and health and education curriculum) will be documented for both control and intervention schools.

In order to deal with the loss of follow-up, some incentives would be provided. All schools completing the trial would be offered all the intervention materials from the project team and they would be honored as “model schools” by the Education Bureau. The class teachers and school doctors involved would receive some financial charges. All the students participated blood sample collection would have a free breakfast and some presents.

### Base line and post baseline data collection

Assessments will be undertaken by the trained project members and experienced research nurses and doctors, according to standard procedures. The assessors wouldn’t be told which schools have been allocated to which arm of the trial after measures have been completed. All measurements were taken in sensitive manners in private rooms in each primary and high school. There were trained research assistants, school doctors and headmasters that remained in the room at all times. Retraining sessions occurred during the data collection period to ensure standard procedures were being employed throughout the measurement. The study equipment was calibrated prior to data collection and weekly thereafter. A summary of all measurements methods are described in Table 2.

**Table 2:** **Outcome measures for the Health Lifestyles Intervention**

**Outcome**	**Time point**		**Device**	**Method**
	**Baseline**	**Post baseline**		
Anthropometric outcomes				
Height	●	●	Portable stadiometer (model TZG, China)	Measured to the nearest mm without shoes.
Weight	●	●	Lever type weight scale (model RGT-140,China)	Measured to the nearest 0.1 kg without shoes and in light clothing
Waist circumference	●	●	Steel tape	Measured to the nearest mm and located at the 1 cm above umbilicus
Hip circumference	●	●	Steel tape	Measured to the nearest mm and located at maximal protrusion of the buttocks
Blood pressure	●	●	Mercury sphygmomanometer, (model XJ1ID, China) and TZ-1 stethophone	Measured from the right arm using a validated mercury sphygmomanometer. The mid upper arm circumference determined cuff size. The cuff was placed approximately 2 cm above the crease of the elbow. The child was seated comfortably for at least 5 minutes prior to the first reading. Blood pressure was measured two times, with one minute between each measurement. Children were asked to remain quiet and to sit still while each reading was being taken. Systolic blood pressure and diastolic blood pressure were recorded.
Behavioral outcomes	●	●	Children Questionnaire; Parent Questionnaire; School Questionnaire	Children under the third grade, both children and parent questionnaires would be filled by parents; Children above the fourth grade, would fill in children questionnaire by themselves in class, instructed by class teacher, and parent questionnaire would be filled by their parents; all the school questionnaire would by filled by school principal or school doctor.
Blood chemical outcomes	●	●		After a 12-h overnight fast, 5 ml venous blood samples were taken from the antecubital vein and collected into EDTA vacuum tubes. Samples were centrifuged at 3000r, aliquoted and stored at −80°C. All biochemical analyses on blood were carried out at a validated biomedical analyses laboratory.
Fasting glucose				Glucose oxidase method
Triglycerides				Enzymatic methods
Cholesterol				Enzymatic methods
HDL-C				Clearance method
LDL-C				Clearance method
Physical fitness outcomes	●	●	Standardized sport equipment	Measured by physical education teachers under the instruction of the project members according to uniform measurement standards

1. Anthropometric: height, weight, waist circumference, hip circumference, skin-fold thickness, blood pressure and five blood sample outcomes were measured at baseline and outcome. About 5% students would be rechecked, and if the error exceeds 10%, all of the students have to be measured again.
2. Behavioral outcome measurements: questionnaires were designed to collect data about obesity related PA and dietary behaviors and possible moderating variables of students. The questionnaires were developed based on the Information, Motivation and Behavioral skills Model [20]; these modified questions, specific to the Health Lifestyles Interventions, have been piloted and revised in early stages of the project and found to be feasible and acceptable to children and teachers in both control and intervention schools. All child-questionnaires (except grade 1–3) are delivered in a class meeting and supported by the class teacher. Both parent and child questionnaire of children grade 1–3 were reported by parents. Trained project members interpreted all of the questionnaires in detail. Appropriate help and guidance would be given by these project members as effectively as possible. The questionnaires would be rechecked by 3% within one week for the same participants. Details of each questionnaire are described below.
  Children Questionnaire: the child-reported questionnaire contained questions under 5 major headings: Background information; Food and diet; Sports and PA; Obesity related knowledge-attitude-practice; School obesity related environment.
  Parent Questionnaire: the Parent/Guardian-reported questionnaire contained questions under 7 major headings: Study child’s birth factors; Family background information; Parental current health; Parental diet; Parental sports and PA; Study child’s PA and diet related parental/community environment; Parents’ obesity related knowledge-attitude-practice.
  School Questionnaire: each participating school was asked to complete a questionnaire which included questions under main headings: Demographics; Health curriculum; School policy; Level of nutritional care; Provision of PA; Community environment.
3. Blood sample measurements: After a 12-h overnight fast, venous blood samples (5 ml) were taken from the antecubital vein and collected into EDTA vacuum tubes. Samples were centrifuged at 3000r, aliquoted and stored at −80°C. All biochemical analyses on blood were carried out at a biomedical analyses company, which is accredited by Peking University. Fasting glucose, triglyceride, total cholesterol, HDL-C, and LDL-C were formally measured.
4. Physical fitness measurements: standing-board jump, 50 meters speed run, 50 meters × 8 shuttle run and run 800/1000 meters would be measured by trained physical education teachers and project members using uniform equipment according to measurement standards.

### Process evaluation

The process evaluation will be conducted in intervention schools twice through the program period to monitor and document the level of implementation of Health Lifestyles Interventions. Moreover, it also aimed to assess the factors that may have influenced its effectiveness and to record stakeholders’ suggestions for future improvements. Based on the steps and principles described in the conceptual framework by Saunders et al. [21], we identified and assessed the process evaluation elements as following: (1) Recruitment/Reach: the recruitment procedure was monitored against the standardized protocol of the study, the proportions and the characteristics of school/teachers/families agreeing to participate in the intervention and completing or dropping out from the intervention were recorded; (2) Dose delivered (completeness): the intensity of actual implementation of the program was assessed; (3) Fidelity: the extent to intervention was implemented as initially planned was assessed;(4) Dose received (exposure and satisfaction): the extent to which children/parents/caregivers/teachers were exposed to the intervention, as well as the degree of their satisfaction with the intervention and material were assessed; (5) Context: several physical (e.g. weather conditions, school infrastructure and curriculum), social (e.g. socioeconomic status of the family, teachers’ health-related behaviors) and contextual (e.g. policies) factors that may have acted as barriers or facilitators to the implementation of the intervention were recorded. Based on the targeted assess element and practical issues [22], the process evaluation methods were designed to include mainly quantitative methods for researchers, teachers and parents: (1) Look up Teachers’ monthly logbooks; (2) Direct regular observation during the implementation of the classroom activities; (3) Regular qualitative interviews of focus groups. Assessed questionnaires and sampling methods have been developed for children, families and schools.

### Statistical analyses

#### Quantitative data analyses

Descriptive statistics were calculated. Between group difference was examined for treatment assignment and overweight status. Differences were tested using independent t-tests for continuous variables (e.g., BMI, blood sample outcomes) and chi-square tests for categorical variables (e.g., overweight, lifestyle factors and obesity related knowledge). Multilevel regression models will be used to test for post-test group differences on the main outcome variables corrected for pre-test measurements. Data will be double entered and cleaned with EpiData 3.0, and managed and analyzed using SPSS 13.0. P < 0.05 is used to assess statistical significance.

#### Qualitative data analyses

All interviews will be audio-taped and transcribed verbatim. Transcribed data will be managed using NVivo 8.0 software which will also support the coding and analytical processes. All of the transcripts will be read and re-read in order to gain an overall understanding of participants' views and experiences. Thematic analysis and comparative analysis will be adopted for the analysis and interpretation.

## Discussion

Present study is the first and largest multi-centered school-based and family-involved children and adolescent obesity intervention study in China. Should the study produce comprehensive results, the intervention strategies would justify a national school-based program to prevent childhood obesity in China.

Data from the 2010 Chinese National Survey on Students’ Constitution and Health (CNSSCH) showed that the overall prevalence for overweight in 2010 was 9.9% and 5.1% for obesity in Chinese school-aged children, with large regional disparities. Besides the greatest increased rates of the epidemic in large coastal cities, similar increases were found in all other regions, including the once poverty stricken rural west. The epidemic in most of the rural areas began after 2000, but has spread swiftly over the last decade [23]. Given the differences in obesity prevalence, food culture (e.g. different appetites, food selection of people living in south and north of China) and environment (e.g. warm temperature enabling more outdoor PA), it is urgent to create obesity intervention in a national level. The Health Lifestyles Interventions was a national health promotion program being designed to benefit and educate all students irrespective of their weight status and socio-demographic backgrounds [10]. Both major and western cities, urban and rural schools were included in this study. They are geographically located in the north, middle and south of China. The participants that enrolled in this project are likely to be largely representative of Chinese children and adolescents.

Health Lifestyles Interventions is based on Social Cognitive Theory and Social-Ecological Model of Health, which emphasizes a dynamic interaction among biological, cultural, and environmental factors over the life course of individuals, families, and communities contributing to the health of populations [24]. It also follow a stepwise approach guided by Precede-Proceed (P-P) Model and Intervention Map (IM), which has been considered to be the best among 10 planning models on usefulness for research and usefulness for practice [25], and initially carries out a systematic review of prospective studies and a secondary data analysis of existing data to identify key behaviors related to obesity in children and adolescent. It then executes focus groups and experts’ seminar to identify the determinants of energy balance-related behaviors. Pilot study of the newly formed interventions involving 4000 students were conducted in 12 schools (Table 1). Based on systematic development and evaluation process, a multi-components (environment, PA, dietary) school-based and family-involved scheme was formed (Table 2). The intervention is designed to instigate change at different levels. Creative delivery methods were used to regulate supportive school and family environment. The importance of PA intensity and dose has been emphasized in several systematic reviews [26]. Students were asked to do moderate to vigorous intensity, age- and space-appropriate exercises at least one hour at each school day. The form of exercises was game, dance or rhythmic gymnastics. Students were also encouraged to develop more forms of exercises they like. Health lifestyle education was mainly involved in dietary intervention. An easy to remember slogan named “52110”, incorporating all the key recommendations about PA and dietary behaviors, has been emphasized throughout health education. In order to dynamically monitor and increase the effectiveness of the interventions, PA and dietary behavioral log was recorded by the focus intervention group students weekly.

School children and adolescent spend a significant amount of their time in school settings. Although formal physical education classes are not the only opportunity for students to engage in PA, such classes offer a regular schedule and structured format both for engaging in PA and for establishing habitual activity early in life. Such a setting provides an ideal and easily accessible environment and has the potential to offer benefits to all children [27], particularly those with no or limited access to play areas outside school [28]. There is good evidence that school-based PA interventions built primarily around promotion of PA are effective in promoting an increase in PA levels and fitness but the evidence for a beneficial effect on obesity levels and cardio-metabolic outcomes remains inconsistent [29]. Four types of outcomes as indicated above were comprehensively measured in our research. Anthropometric outcome, especially BMI, is the most reported measurement of body composition [30]. However, the evidence for school-based intervention programs in reducing skin-fold thickness is consistent than BMI and percent body fat [31]. Research results showed that PA interventions can improve HDL cholesterol levels and blood pressure in the short term but the evidence for triglycerides, LDL–C and total cholesterol levels remains inconclusive [26,32]. Few studies reported on behavioral outcomes (e.g. PA, eating and sedentary behaviors, teachers’ or parental knowledge, beliefs and attitudes, school environment), valid and reliable tools are needed for the measurements [29]. Measurements of physical fitness were varied among studies, standing-board jump, 50 meters speed run, 50 meters × 8 shuttle run and run 800/1000 meters were measured in our study. Results from several reviews showed that large, higher quality RCTs showed an effectiveness of the intervention on improving fitness and the effectiveness may be determined by the cumulative amount of physical activity performed [29]. As no single intervention will fit all schools and populations and the inconsistent outcomes of different studies, further high-quality research needs to focus on identifying specific program characteristics predictive of success.

## Abbreviations

BMI: Body mass index
PA: Physical activity
RCT: Randomized Controlled Trial
HDL-C: High-density lipoprotein cholesterol
LDL-C: Low-density lipoprotein cholesterol
CNSSCH: Chinese National Survey on tudents’ Constitution and Health
P-P: Precede-Proceed
IM: Model and Intervention Map
